# Diurnal Variability of Platelet Aggregation in Patients with Myocardial Infarction Treated with Prasugrel and Ticagrelor

**DOI:** 10.3390/jcm11041124

**Published:** 2022-02-21

**Authors:** Piotr Adamski, Malwina Barańska, Małgorzata Ostrowska, Wiktor Kuliczkowski, Katarzyna Buszko, Katarzyna Kościelska-Kasprzak, Bożena Karolko, Andrzej Mysiak, Jacek Kubica

**Affiliations:** 1 Department of Cardiology and Internal Medicine, Collegium Medicum, Nicolaus Copernicus University, 85-094 Bydgoszcz, Poland; baranskamalwina@gmail.com (M.B.); m.ostrowska@cm.umk.pl (M.O.); jkubica@cm.umk.pl (J.K.); 2 Institute for Heart Diseases, Wroclaw Medical University, 50-556 Wroclaw, Poland; wiktor.kuliczkowski@umed.wroc.pl (W.K.); bozena.karolko@umw.edu.pl (B.K.); andrzej.mysiak@umw.edu.pl (A.M.); 3 Department of Theoretical Foundations of Biomedical Science and Medical Informatics, Collegium Medicum, Nicolaus Copernicus University, 87-067 Bydgoszcz, Poland; buszko@cm.umk.pl; 4 Department and Clinic of Nephrology and Transplantation Medicine, Wroclaw Medical University, 50-367 Wroclaw, Poland; katarzyna.koscielska-kasprzak@umw.edu.pl

**Keywords:** myocardial infarction, platelet aggregation variability, prasugrel, ticagrelor

## Abstract

Background: Contemporary antiplatelet treatment in acute myocardial infarction (AMI) is based on one of two P2Y12 receptor inhibitors, prasugrel or ticagrelor. The aim of this study was to compare diurnal variability of platelet reactivity between patients receiving prasugrel and ticagrelor during the initial phase of maintenance treatment after AMI. Methods: It was a prospective, two-center, pharmacodynamic, observational study. Blood for platelet testing was sampled at four time points on day four after AMI (8:00, 12:00, 16:00, 20:00). Diurnal variability of platelet reactivity was expressed as a coefficient of variation (CV) of the above-mentioned measurements. Results: 73 invasively-treated patients were enrolled (ticagrelor: *n* = 47, prasugrel: *n* = 26). CV was greater in patients treated with ticagrelor compared with prasugrel according to a VASP assay (47.8 [31.6–64.6]% vs. 21.3 [12.9–25.5]%, *p* < 0.001), while no statistical differences were detected when the CVs of platelet aggregation according to Multiplate were compared between ticagrelor- and prasugrel-treated patients. Ticagrelor-treated patients showed more pronounced platelet inhibition than prasugrel at 16:00 and 20:00 (VASP_16:00_: 20.6 ± 15.0 vs. 24.9 ± 12.8 PRI, *p* = 0.049; VASP_20:00_: 18.6 ± 17.7 vs. 26.0 ± 11.7 PRI, *p* = 0.002). Conclusions: Ticagrelor shows greater diurnal variability in platelet aggregation than prasugrel during the initial maintenance phase of AMI treatment, and this is due to the continuous increase of platelet inhibition after the morning maintenance dose. Both drugs provide an adequate antiplatelet effect early after AMI. Evaluation of the clinical significance of these findings warrants further investigation.

## 1. Introduction

Acute myocardial infarction (AMI) is a life-threatening condition caused by prolonged myocardial ischemia. The incidence and mortality of AMI largely depends on the general cardiovascular risk of certain populations [1]; nevertheless, it remains one of the leading causes of morbidity and mortality worldwide. The yearly incidence of AMI in the United States is approximately 600 cases per 100,000 people, which accounts for 1.5 million cases annually [2]. In the European Union alone, AMI is responsible for over 200,000 deaths each year [3].

In the vast majority of cases, AMI occurs against the background of coronary atherosclerosis, and rupture or erosion of atherosclerotic plaque with subsequent formation of intracoronary thrombus is the most common underlying mechanism [4]. Restoration of patency of the culprit vessel is the cornerstone of AMI treatment, as it mitigates ischemia and prevents further myocardial necrosis. Coronary revascularization is usually obtained either with percutaneous coronary intervention (PCI) or coronary artery bypass grafting [5].

Dual antiplatelet treatment (DAPT), combining aspirin and one of the P2Y12 receptor antagonists, is an integral part of AMI management. DAPT in this setting is necessary to limit the excessive platelet activation that occurs during and after the acute coronary event. Platelet inhibition curtails a pro-thrombotic state and reduces the incidence of recurrent ischemic events [5]. Use of DAPT is also essential to prevent stent thrombosis, a potentially lethal complication of PCI.

Three oral P2Y12 receptor inhibitors are recommended for the treatment of AMI: clopidogrel, prasugrel and ticagrelor. In the majority of patients, prasugrel and ticagrelor are preferred over clopidogrel due to improved cardiovascular outcomes [5]. The clinical superiority of prasugrel or ticagrelor above each other is debatable, as reports on this matter are not consistent [6,7,8,9,10,11]. The ISAR-REACT 5 trial, the only available randomized, clinical, head-to-head study, showed a reduction of composite ischemic endpoint (death, AMI, stroke) with prasugrel vs. ticagrelor in ACS patients [6]. However, the study had limitations that impede interpretation of its results and applicability [10]. On the other hand, data from registries and observational studies are neutral [11] or show ischemic benefit when ticagrelor is used instead of prasugrel in patients with ACS or AMI [7,8].

**Figure 1:**
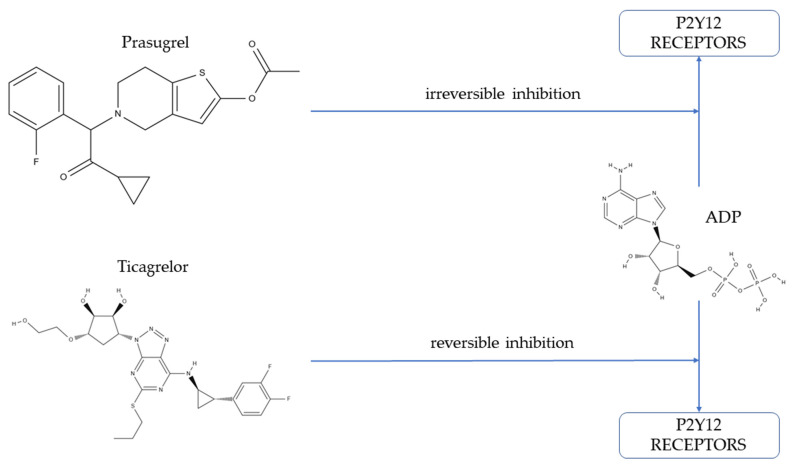
Mechanism of action of prasugrel and ticagrelor. ADP: adenosine-5′-diphosphate.

During the first hours of AMI, the pharmacodynamics of prasugrel and ticagrelor are similar, and both drugs exert a comparable antiplatelet effect early after the loading dose [12]. During the maintenance phase, both drugs provide stronger platelet inhibition than clopidogrel [13,14]. Prasugrel and ticagrelor inhibit adenosine-5′-diphosphate (ADP)-induced platelet aggregation via interaction with Gi-coupled ADP P2Y12 receptors, which play a crucial role in activation of thrombocytes (Figure 1) [15].

Several important pharmacological differences exist between these two P2Y12 receptor antagonists. Prasugrel is a pro-drug that requires hepatic activation to exert its antiplatelet effect. Active metabolite of prasugrel irreversibly binds closely to the ADP-binding site of the P2Y12 receptor, leading to inhibition of platelet aggregation, lasting for the whole lifespan of the platelet [16,17,18]. Ticagrelor is a cyclopentyl-triazolo-pyrimidine that is an active drug, which also undergoes hepatic metabolism and has one active metabolite [19]. Both ticagrelor and its active metabolite reversibly inhibit P2Y12 receptors independently from the ADP binding site [20,21]. Another important difference between prasugrel and ticagrelor is that the prasugrel maintenance dose is administered once daily, while ticagrelor requires dosing every 12 h [22].

Circadian variation in the occurrence of myocardial infarction has been well documented, showing an increased risk of AMI in the morning, which among other things has been attributed to increased platelet reactivity during this part of the day [23]. This daily fluctuation in platelet activity is also observed in AMI patients treated with clopidogrel [24]. Platelet reactivity in healthy volunteers receiving ticagrelor seems to follow this diurnal pattern as well [25]. Moreover, a morning peak of stent thrombosis has been reported in patients with acute coronary syndromes (ACS) treated with thienopyridines, clopidogrel or prasugrel [26]. Additionally, the highest risk of thrombotic complications after ACS is observed during the first month after the acute coronary event, highlighting how important adequate antiplatelet therapy is in this early phase of treatment [27,28].

The majority of pharmacodynamic studies evaluating P2Y12 antagonists performed so far have focused either on the initial hours of antiplatelet treatment during ACS or the stable, chronic phase [12,13,14,29,30,31]. The first period is pivotal, especially in invasively- treated patients, in whom a fast and potent antiaggregatory effect is necessary to avoid periprocedural thrombotic complications and early recurrence of myocardial ischemia. The latter period, usually evaluated 4 weeks after ACS or later, is important in terms of chronic response to antiplatelet agents. The first several days after AMI are less explored but nonetheless should also be considered important. During this period of time, platelet aggregation remains elevated compared to the baseline, and patients are often not entirely stabilized. In addition, the risk of thrombotic events is still reasonably high and has not reached a stable plateau yet [27,28,32]. Moreover, up-to-date variation in daily platelet reactivity during the initial phase of maintenance treatment after AMI has never been directly compared between prasugrel- and ticagrelor-treated patients.

The aim of this study was to compare circadian fluctuations in on-treatment platelet reactivity following the standard maintenance doses of prasugrel and ticagrelor evaluated on day four after invasively-treated AMI.

## 2. Materials and Methods

### 2.1. Study Design and Population

The DRAGON (Daily Variability of Platelet Aggregation in Patients With Myocardial Infarction Treated With Prasugrel and Ticagrelor) trial was a prospective, phase IV, two-center, pharmacodynamic, observational study (ClinicalTrials.gov Identifier: NCT03454841). The trial was initially registered as a randomized study; however, its design was changed to observational before the first patient was enrolled.

The study sites were: (1) Department of Cardiology and Internal Medicine, Collegium Medicum, Nicolaus Copernicus University, Bydgoszcz, Poland; (2) Department and Clinic of Cardiology, Wrocław Medical University, Wrocław, Poland. The study was approved by the local ethics committee (The Ethics Committee of Nicolaus Copernicus University in Toruń, Collegium Medicum in Bydgoszcz; study approval reference number KB 101/2016) and was conducted in accordance with the principles contained in the Declaration of Helsinki and Good Clinical Practice guidelines. All enrolled patients provided written informed consent to participate in the trial.

Inclusion criteria were: (1) age between 18 and 75 years; (2) diagnosed AMI (ST-elevation myocardial infarction [STEMI] or non-ST elevation myocardial infarction [NSTEMI]); (3) index event treated with PCI. Exclusion criteria included: (1) treatment with any P2Y12 receptor inhibitor within 14 days before the study enrollment; (2) hypersensitivity to prasugrel or ticagrelor; (3) contraindications for prasugrel or ticagrelor; (4) current treatment with oral anticoagulant or chronic therapy with low-molecular-weight heparin; (5) active bleeding; (6) history of ischemic stroke or transient ischemic attack; (7) history of intracranial hemorrhage; (8) history of gastrointestinal bleeding within last 30 days; (9) history of moderate or severe hepatic impairment; (10) history of major surgery or severe trauma (within 3 months); (11) patient on dialysis; (12) infection or inflammatory state; (13) therapy with strong CYP3A inhibitors or inducers; (14) body weight below 60 kg. The diagnosis of STEMI or NSTEMI was established according to the Third Universal Definition of Myocardial Infarction, which was the up-to-date version of the AMI definition when the trial was designed and initiated [33].

All study participants received orally a loading dose of 300 mg aspirin, and either 60 mg of prasugrel or 180 mg of ticagrelor. Choice of P2Y12 receptor inhibitor (prasugrel vs. ticagrelor) was left to the physician’s discretion. All study participants were treated with PCI (either drug-eluting stent implantation or drug-coated balloon angioplasty) for the index AMI. Medical treatment was administered in line with the current ESC guidelines [22,34,35]. After receiving a loading dose of ticagrelor or prasugrel, study participants continued on a maintenance dose of 10 mg prasugrel once daily or 90 mg ticagrelor twice daily. All patients received aspirin, beta-blockers, statins, and angiotensin-converting enzyme inhibitors or angiotensin II receptor blockers, unless they were contraindicated.

### 2.2. Endpoints

The pre-defined primary endpoint of this study was circadian variability of platelet reactivity assessed on the fourth day after AMI using a Vasodilator-Associated Stimulated Phosphoprotein (VASP) assay. The co-primary endpoint was diurnal variability of platelet reactivity evaluated with a Multiplate platelet function test parallel to VASP. The variability of platelet aggregation was expressed as the coefficient of variation (CV), showing the standard deviation of platelet aggregation relative to the mean, calculated individually for each patient based on platelet function test measurements at 8:00, 12:00, 16:00 and 20:00 on the fourth day after AMI. Secondary endpoints included incidence of high platelet reactivity (HPR) on the fourth day after AMI evaluated at 8:00, 12:00, 16:00 and 20:00 with both VASP and Multiplate. Additionally, we compared on-treatment platelet reactivity between the two groups in the aforementioned time points using both platelet function tests.

### 2.3. Blood Collection

Samples for the pharmacodynamic evaluation were collected using a venous catheter (18G) inserted into one of the forearm veins, and the first 5 mL portion of blood was discarded to prevent spontaneous platelet activation. Blood was drawn at four pre-defined time points on day four after AMI (8:00—directly before the morning maintenance dose of ticagrelor or prasugrel, 12:00, 16:00, 20:00—directly before the evening maintenance dose of ticagrelor).

### 2.4. Assessment of Platelet Function

Platelet function testing was carried out in all study participants using VASP assay (Biocytex, Inc., Marseille, France) and Multiplate analyzer (Roche Diagnostics International Ltd., Rotkreuz, Switzerland), as previously described [36,37]. In line with the recommended thresholds, HPR was defined as the platelet reactivity index (PRI) > 50% for the VASP assay, and > 46 units (U) for Multiplate [38]. The laboratory staff performing the platelet function tests was blinded to the received antiplatelet treatment.

### 2.5. Sample Size Calculation and Statistical Analysis

The sample size calculation was based on VASP assay-derived pharmacodynamic data obtained from the internal pilot study that consisted of the first 24 enrolled patients, including 15 subjects treated with ticagrelor and 9 receiving prasugrel. Based on these results, using the *t*-test for independent variables and assuming a two-sided alpha value of 0.02, we calculated that inclusion of at least 46 and 26 patients receiving ticagrelor and prasugrel, respectively, would provide a 98% chance of demonstrating a significant difference between the study groups.

Statistical calculations were performed using the Statistica 13 package (StatSoft, Tulsa, OK, USA), Matlab 2017b (Matlab and Statistics Toolbox Release 2017, The MathWorks Inc., Natick, MA, USA) and R version 3.5.0 (The R Foundation, Vienna, Austria). Data for pharmacodynamic variables were presented as means with standard deviations. Data for CV, age, body mass index, laboratory test results and left ventricular ejection fraction were presented as medians and interquartile ranges. Continuous variables were compared between the study groups with the Student’s *t*-test and Mann–Whitney U test, depending on the presence or absence of the normal distribution (as assessed by the Shapiro–Wilk test). Comparisons between categorical variables were performed by the chi-square test, with Yates’s correction if necessary, or using Fisher’s exact test. CV was calculated individually for all patients based on their consecutive platelet function test measurements. The mathematical formula for calculating CV was:

(1) $$\mathrm{CV}=\frac{\mathrm{standard}\;\mathrm{deviation}}{\mathrm{mean}}\times 100\%.$$

In order to model the CV of platelet aggregation, a mixed model with random effects was fitted to the data, and the type of antiplatelet agent, age, gender, body mass index, obesity, type of AMI, hypertension, diabetes mellitus, hyperlipidemia, smoking status, prior coronary artery disease, prior PCI, left ventricular ejection fraction, creatinine, glomerular filtration rate, brain natriuretic peptide, uric acid, hemoglobin, red blood cells, hematocrit, white blood cells, platelets, and mean platelet volume were separately included in the model as covariates. In all cases, two-sided *p* values < 0.05 were considered significant.

## 3. Results

### 3.1. Baseline Characteristics

Overall, 73 patients with invasively treated AMI were enrolled into the trial, of which 47 were treated with ticagrelor and 26 received prasugrel. Mean age of the study participants was 56.2 ± 9.8 years old. They were mainly men (72.6%) and were hospitalized due to STEMI rather than NSTEMI (79.5% vs. 20.5%, respectively). Baseline characteristics were well balanced between patients treated with ticagrelor and those receiving prasugrel (Table 1).

### 3.2. Pharmacodynamic Outcomes

The median of individual CVs of on-treatment platelet reactivity evaluated with VASP on day 4 after AMI was significantly greater in patients treated with ticagrelor compared with prasugrel (47.8 [31.6–64.6]% vs. 21.3 [12.9–25.5]%, *p* < 0.001). No statistical differences were detected when the CVs of platelet aggregation according to Multiplate were compared between ticagrelor- and prasugrel-treated AMI patients (23.1 [16.7–35.0]% vs. 19.4 [11.7–30.8]%, *p* = 0.20).

The secondary endpoint, which was the rate of HPR, did not differ between the study groups in the morning or at any later of the examined time points according to both platelet function tests. Generally, HPR rates were low in most of the time points, showing adequate platelet inhibition throughout the day with both P2Y12 receptor antagonists (Table 2). Nevertheless, elevated occurrence of HPR was observed in both groups at 20:00 when platelet reactivity was evaluated with Multiplate (ticagrelor vs. prasugrel: 21.3% vs. 15.4%). This was not observed with VASP (ticagrelor vs. prasugrel: 6.4% vs. 3.9%).

**Figure 2:**
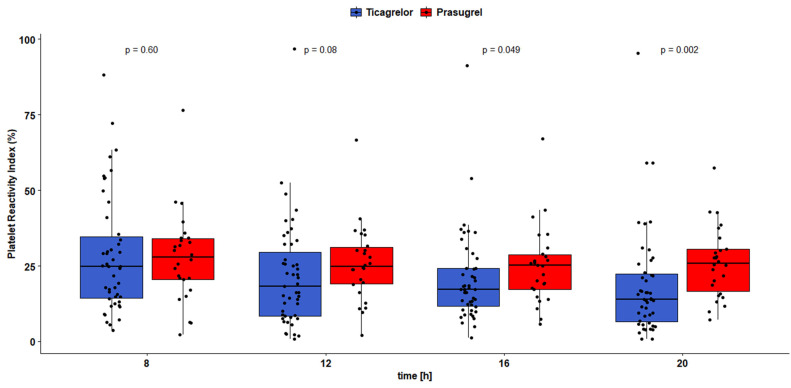
Platelet reactivity according to VASP assay.

**Figure 3:**
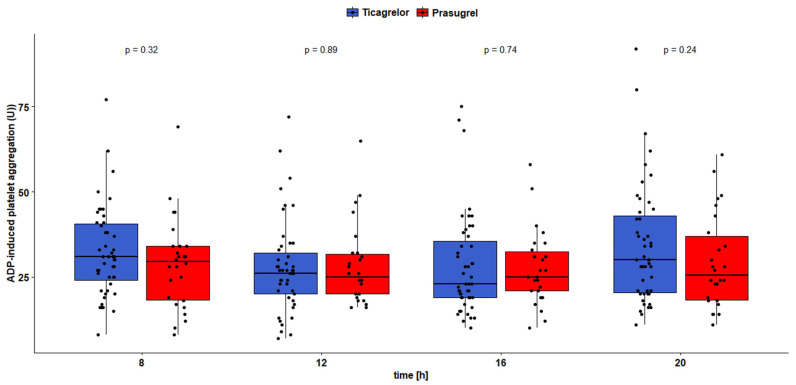
Platelet reactivity according to Multiplate assay.

No differences in platelet reactivity were found between the groups when it was evaluated immediately before the morning P2Y12 receptor inhibitor maintenance dose at 8:00 or 4 h later at 12:00. Patients receiving ticagrelor presented more pronounced platelet inhibition than those on prasugrel according to VASP at 16:00 and 20:00 (VASP_16:00_: 20.6 ± 15.0 vs. 24.9 ± 12.8 PRI, *p* = 0.049; VASP_20:00_: 18.6 ± 17.7 vs. 26.0 ± 11.7 PRI, *p* = 0.002; Figure 2). No differences were observed in platelet inhibition in these time points according to Multiplate (Figure 3). 

We fitted 24 one-dimensional models with available variables, and only the administered P2Y12 receptor antagonist had a significant influence on the CV of daily platelet reactivity according to VASP, increasing it by 28.2% (*p* < 0.001) when ticagrelor was used (Table 3). The analogous model performed for platelet reactivity evaluated with Multiplate revealed that daily fluctuation of platelet aggregation had decreased by 3.2%, 1.1% and 0.5%, with an increase of hemoglobin concentration by every g/dL (*p* = 0.028), hematocrit by 1% (*p* = 0.047) and mean platelet volume by every fL (*p* = 0.03), respectively (Table 3).

### 3.3. Adverse Events

The study population was monitored for adverse bleeding and thrombotic events; however, none was observed within the observation period, which lasted 12 h between the first and the last blood sampling (from 8:00 till 20:00 on day four after AMI).

## 4. Discussion

To our knowledge, the current trial is the first study directly comparing daily profiles of the antiaggregatory action of prasugrel and ticagrelor in the initial phase of maintenance treatment after myocardial infarction.

Prasugrel and ticagrelor are the first-line P2Y12 receptor inhibitors in patients with ACS, including those with AMI. Because of their clinical superiority over clopidogrel shown in the landmark trials, both agents are preferred in the majority of ACS patients [5,22,27,28]. DAPT with prasugrel or ticagrelor should be administered for 12 months after ACS, unless contraindications or excessive bleeding risk exists. Potential shortening or extension of DAPT should be based on individual ischemic and bleeding risk [22]. In contrast to ticagrelor, prasugrel should not be used in ACS patients designated to conservative treatment and patients in whom coronary anatomy is not known. Neither of the described P2Y12 receptor antagonists should be used in ACS patients with indications for chronic oral anticoagulation [5,22]. Prasugrel or ticagrelor may also be considered instead of clopidogrel in specific high-risk situations of elective stenting [22].

Prasugrel (2-acetoxy-5-(α-cyclopropylcarbonyl-2-fluorobenzyl)-4,5,6,7-tetrahydrothieno [3,2-c]pyridine) is a thienopyridine pro-drug (Figure 1) that requires hepatic activation to exert its antiplatelet effect [16]. The biotransformation of prasugrel into its active metabolite (R-138727) involves rapid de-esterification to inactive metabolite (R-95913), followed by cytochrome P450-mediated formation of R-138727. Active metabolite of prasugrel is formed primarily by CYP3A4/5 and CYP2B6, with a smaller contribution from CYP2C9 and CYP2C19 [39]. R-138727 irreversibly binds closely to the ADP-binding site of P2Y12 receptor, leading to inhibition of platelet aggregation lasting for the whole lifespan of the platelet [16,17,18]. At the initiation of treatment with prasugrel, a loading dose of 60 mg should be administered, followed by a maintenance dose of 10 mg once daily, unless a patient weighs <60 kg or is ≥75 years old, in which case a reduced dose of 5 mg once daily is recommended [5]. Some trials evaluated clinical outcomes with a lower maintenance dose of 3.75 mg once daily. A meta-analysis of these studies, performed in patients (*n* = 32,951) treated with PCI for ACS or chronic coronary syndrome, has shown that low-dose prasugrel (3.75 mg in Asian or 5 mg in European patients) was associated with a 20% lower risk of major adverse cardiovascular events (odds ratio [OR] 0.80, 95% confidence interval [CI] 0.67–0.97) compared with clopidogrel, which was mainly driven by reduction of AMI (OR 0.74, 95% CI 0.56–0.98). At the same time, patients receiving reduced-dose prasugrel had a higher risk of minor bleeding (OR 1.73, 95% CI 1.25–2.41) [40]. Of note, in order to maintain the homogeneity of the study population, the exclusion criteria for the current study did not allow enrolment of patients with an indication for a reduced maintenance dose of prasugrel.

Ticagrelor (1S,2S,3R,5S)-3-[7-[[(1R,2S)-2-(3,4-difluorophenyl)cyclopropyl]amino]-5-(propylthio)-3H-1,2,3-triazolo [4,5-d]pyrimidin-3-yl]-5-(2-hydroxyethoxy)-1,2-cyclopentanediol) is a cyclopentyl-triazolo-pyrimidine (Figure 1) that is an active drug which also undergoes hepatic metabolism. CYP3A4 and CYP3A5 enzymes are mainly responsible for the formation of 10 known metabolites of ticagrelor, and only one (AR-C124910XX) of them shows antiplatelet potential. The active metabolite of ticagrelor is formed through O-deethylation and exerts comparable platelet inhibition as the parent drug [19,41]. Both ticagrelor and its active metabolite antagonize ADP-induced platelet aggregation by binding to the P12Y12 receptor in a reversible manner and independently from ADP [20,21]. At the beginning of treatment with ticagrelor, a loading dose of 180 mg should be administered, followed by a maintenance dose of 90 mg twice daily. In selected, high ischemic risk patients with prior AMI ticagrelor, 60 mg twice daily may be used together with aspirin beyond 12 months of standard DAPT [5]. In pursuing optimalization of antiplatelet treatment after ACS, a wide range of reduced doses have been examined (1 × 90 mg, 2 × 60 mg, 1 × 60 mg, 2 × 45 mg, 2 × 22.5 mg) [42]. Importantly, the majority of studies evaluating doses below 2 × 60 mg were performed in Asian patients only and were mainly pharmacodynamic trials. Interestingly, even reduced maintenance doses of ticagrelor provide adequate platelet inhibition, which is greater than in clopidogrel-treated patients. De-escalation of the ticagrelor dose shows a propensity towards a reduced rate of bleeding and non-bleeding adverse events [42].

The current trial was designed to explore and compare pharmacodynamics of the guideline-recommended treatment used in everyday clinical practice; thus, standard maintenance doses of prasugrel (10 mg once daily) and ticagrelor (90 mg twice daily) were applied in all participants [5]. Moreover, the observation period was day 4 after AMI, which still has to be considered an acute phase of AMI, while all de-escalation strategies focus rather on the period after 1 month post-AMI, when the baseline pro-thrombotic state stabilizes [31,40,42,43].

Ticagrelor and prasugrel exert more potent antiplatelet action compared with clopidogrel, both in the acute and maintenance phase, which translates into improved cardiovascular outcomes [13,14]. In patients with AMI, both agents show similar pharmacodynamics following a loading dose, with no significant differences in platelet inhibition during the first 12 h [12]. Even though prasugrel and ticagrelor are characterized by a rapid onset of antiplatelet action, in the acute setting they may require 3 to 5 h to reach platelet inhibition below HPR [12]. Similar pharmacodynamic observations were made during the first 3 days of treatment in patients after cardiac arrest due to AMI who were treated with mild therapeutic hypothermia [44]. In this specific subpopulation of AMI, no differences in platelet inhibition were detected on day 1, 2 or 3 of antiplatelet treatment between the prasugrel- and ticagrelor-treated patients. Importantly, even in this high-risk group, HPR rates were negligible [44]. Kerneis et al. performed an extensive comparison of antiplatelet effects obtained with prasugrel and ticagrelor 30 days after invasively-treated ACS. Platelet function was evaluated in 118 patients using 3 assays. In this trial, ticagrelor produced stronger platelet inhibition compared with prasugrel; however, this was observed only with a VerifyNow assay (20.91 ± 4.59 PRU vs. 43.50 ± 6.98 PRU, *p* = 0.008) and was not confirmed by VASP or light transmittance aggregometry (*p* = 0.09 for both tests) [45]. These direct comparisons of both P2Y12 receptor inhibitors indicate the comparable antiplatelet potency of prasugrel and ticagrelor during the first month after ACS.

Our study demonstrates that ticagrelor shows greater diurnal variability in platelet inhibition during the initial days after AMI compared with prasugrel. The CV of platelet reactivity according to VASP evaluated individually for all study participants was greater by 26.5% in ticagrelor-treated patients than in those receiving prasugrel (*p* < 0.001). This was confirmed by one-dimensional mixed modeling with random effects, which indicated a 28.2% greater CV of diurnal VASP platelet aggregation when ticagrelor was used (*p* < 0.00001). Administration of ticagrelor was responsible for 31% of the observed variability of CV in the created model. Importantly, this more pronounced variation in on-treatment circadian platelet reactivity resulted from the stable and continuous increase of platelet inhibition in the consecutive time points after the morning maintenance dose, as shown with VASP (Figure 2). In the examined period of time, AMI patients administered with prasugrel showed a more consistent pattern of platelet inhibition with significantly smaller alternation during the day.

The occurrence of HPR in our trial did not differ significantly between patients treated with ticagrelor and prasugrel, regardless of the time point or utilized platelet function assay. Nevertheless, depending on a sampling point, we observed a range of HPR occurrences among study participants (3.9–21.3%), suggesting a certain interindividual diurnal variability in response to each P2Y12 receptor inhibitor. Despite that, mean platelet reactivity evaluated directly before the morning maintenance dose on day four after AMI was substantially below VASP and Multiplate thresholds for HPR in both study groups, showing satisfactory platelet inhibition prior to the morning maintenance dose. Additionally, mean platelet reactivity in both groups in all of the remaining time points was also considerably below the HPR threshold, irrespective of the platelet function test that was applied (Figure 2 and Figure 3). According to platelet testing with VASP, patients receiving ticagrelor showed a constant decrease in platelet reactivity throughout the day, which resulted in a significantly stronger antiplatelet effect 8 and 12 h after the morning maintenance dose (at 16:00 and 20:00) compared with patients on prasugrel.

In healthy volunteers, platelet reactivity on ticagrelor remains higher in the morning, which resembles the circadian variability observed in untreated subjects [25]. This observation appears to be true also for AMI patients treated with ticagrelor, as in our trial they had the highest platelet reactivity at 8:00 in the morning according to the VASP assay. When assessed with Multiplate, patients receiving ticagrelor have shown a slight rise of platelet reactivity directly before the evening maintenance dose, which was not seen with VASP. Nevertheless, ticagrelor produced a satisfactory antiplatelet effect during the whole day, and, according to VASP, showed a continuous increase in diurnal platelet inhibition after the morning maintenance dose.

Previous studies documented an increased risk of AMI in the morning, which was hypothesized to be linked with elevated platelet reactivity in the morning hours [23]. A post hoc analysis of the TRITON-TIMI 38 trial [27] showed a higher rate of stent thrombosis in the early part of the day in patients undergoing dual antiplatelet therapy with aspirin and prasugrel [26]. However, a sub-study of the TROPICAL-ACS trial showed that prasugrel-treated ACS patients do not display diurnal variability or a peaking of platelet reactivity in the morning, when evaluated 14 days after the acute coronary event. In the same study, patients receiving clopidogrel showed significant diurnal variability of platelet aggregation, with a peaking of platelet reactivity in the early morning (5–10 a.m.) [46]. In line with these results, in our trial we have seen very modest alterations in platelet inhibition exerted by prasugrel. Similarly to ticagrelor, prasugrel produced sufficient platelet inhibition during the whole examined period, including the morning hours. However, prasugrel-treated patients demonstrated a weaker antiplatelet effect compared with those on ticagrelor at 16:00 and 20:00.

In spite of having a comparable mean elimination half-life (approximately 13–15 h), ticagrelor requires dosing twice daily, while prasugrel is administered only once per day, and the antiplatelet effect of ticagrelor is almost completely abolished 3–5 days after the last dose, whereas platelet inhibition with prasugrel extends to the lifespan of the platelet, lasting up to 7 days [18,19,22]. Interestingly, circadian platelet aggregation in prasugrel-treated patients is invariable and appears not to follow the daily platelet reactivity pattern, most likely due to the irreversible and potent blockade of platelet P2Y12 receptors. On the other hand, overlapping of the diurnal platelet reactivity pattern and reversible nature of the P2Y12 receptor blockade serves as a logical justification for the continuous daily increase of platelet inhibition in AMI patients receiving ticagrelor.

Our post hoc one-dimensional modeling suggested a modest, negative effect of hemoglobin concentration, hematocrit and mean platelet volume on the CV of platelet aggregation assessed with Multiplate (Table 3). Despite reaching statistical significance, these variables were responsible only for 6–7% of circadian variability in platelet inhibition and the clinical significance of these findings remains vague. Currently available data suggest that lower concentrations of hemoglobin are associated with a higher rate of HPR and an increased rate of recurrent ACS in patients treated with ticagrelor [47]. In ticagrelor-treated patients, mean platelet volume does not influence the risk of HPR; however, it is related with an increased rate of cardiovascular events in ACS patients [48,49]. Studies on potential correlations between prasugrel and the above-mentioned laboratory variables are lacking.

Despite the observed differences in circadian on-treatment platelet reactivity pattern, it has to be underlined that both recommended P2Y12 receptor inhibitors provided adequate platelet inhibition. Importantly, ticagrelor and prasugrel exerted a comparable and satisfactory antiplatelet effect during the morning hours, when a peak of adverse thrombotic events is observed and potent antiaggregatory action is crucial.

### Study Limitations

Admittedly, differences between AMI patients treated with ticagrelor and prasugrel were shown only with one of the two used platelet function tests (VASP—a pre-defined, co-primary endpoint). Nevertheless, the sample size calculation was based on VASP pharmacodynamic data, which may explain lack of differences seen with Multiplate. By definition, the current trial was a pharmacodynamic study; thus, the sample size was insufficient to evaluate any clinical endpoints. Our trial assessed antiplatelet effects of ticagrelor and prasugrel without evaluation of their pharmacokinetics. Lastly, the choice of antiplatelet treatment was left to the physician’s discretion and was not allocated randomly, although no differences in baseline characteristics were detected between the study groups.

## 5. Conclusions

The DRAGON study is the first direct comparison of diurnal pharmacodynamic profiles of prasugrel and ticagrelor in the early maintenance phase of treatment after AMI. Ticagrelor displays greater diurnal variability in on-treatment platelet aggregation than prasugrel due to the continuous increase of platelet inhibition after the morning maintenance dose. Importantly, despite these differences, both drugs provide an adequate antiplatelet effect.

It is not clear whether the very stable and peakless, but slightly less pronounced platelet inhibition obtained with prasugrel is more beneficial than the more potent antiplatelet action of ticagrelor, which increases between doses. Available clinical data also do not provide apparent answers as to which of the tested P2Y12 antagonists should be preferred in the general ACS population. Further studies are needed to investigate whether prasugrel or ticagrelor could be more advantageous in specific clinical scenarios.

## Figures and Tables

**Table 1:** Baseline characteristics of trial participants.

Variable	Ticagrelor (*n* = 47)	Prasugrel (*n* = 26)	*p* Value
Age, years	59 [51–63]	58 [48–63]	0.95
Female	16 (34%)	4 (15%)	0.15
Body mass index, kg/m^2^	27.9 [25.5–30.1]	27.8 [24.9–29.4]	0.91
STEMI	37 (79%)	21 (81%)	0.92
Hypertension	30 (64%)	16 (62%)	0.95
Diabetes mellitus	9 (19%)	3 (12%)	0.31
Hyperlipidemia	44 (94%)	20 (77%)	0.06
Current smoker	29 (62%)	20 (77%)	0.28
Prior CAD	6 (13%)	7 (27%)	0.23
Prior AMI	5 (11%)	4 (15%)	0.72
Prior PCI	5 (11%)	4 (15%)	0.72
Prior CABG	1 (2%)	0	n/a
Peripheral arterial disease	3 (6%)	1 (4%)	0.55
Prior heart failure	1 (2%)	2 (8%)	0.29
COPD	3 (6%)	0	n/a
Chronic renal disease	1 (2%)	2 (8%)	0.29
Gout	0	2 (8%)	0.12
LVEF at discharge, %	45 [40–50]	47 [38–50]	0.69
Creatinine, mg/dL	0.83 [0.74–1.02]	0.84 [0.77–1.02]	0.73
GFR, mL/min	86 [72–97]	74 [60–95]	0.73
CRP, mg/L	8.6 [3.8–24.0]	10.2 [4.0–20.3]	0.59
BNP, pg/mL	120 [72–185]	107 [79–234]	0.93
Uric acid, mg/dL	5.9 [4.8–6.4]	5.7 [5.1–6.4]	0.67
Hemoglobin, g/dL	14.9 [13.8–15.5]	14.9 [14.4–15.9]	0.63
RBC, 10^12^/L	4.8 [4.8–5.1]	4.8 [4.5–5.3]	0.52
HCT, %	44 [41–46]	44 [41–47]	0.66
WBC, 10^9^/L	10.5 [8.6–13.4]	10.6 [7.7–13.3]	0.60
PLT, 10^9^/L	239 [203–279]	256 [214–301]	0.32
MPV, fL	10.9 [10.2–11.4]	10.7 [9.7–11.3]	0.29

Data are shown as median [interquartile range] or number (%). AMI: acute myocardial infarction; BNP: brain natriuretic peptide; CABG: coronary artery bypass graft; CAD: coronary artery disease; COPD: chronic obstructive pulmonary disease; CRP: C-reactive protein; GFR: glomerular filtration rate; HCT: hematocrit; LVEF: left ventricular ejection fraction; MPV: mean platelet volume; n/a: not available; PCI: percutaneous coronary intervention; PLT: platelets; RBC: red blood cells; STEMI: ST-elevation myocardial infarction; WBC: white blood cells.

**Table 2:** Incidence of high platelet reactivity.

Sampling Time Point (Hour)	VASP			Multiplate		
	Ticagrelor (*n* = 47)	Prasugrel (*n* = 26)	*p* Value	Ticagrelor (*n* = 47)	Prasugrel (*n* = 26)	*p* Value
08:00	8 (17.0%)	1 (3.9%)	0.20	5 (10.6%)	2 (7.7%)	0.52
12:00	2 (4.3%)	1 (3.9%)	0.71	4 (8.5%)	3 (11.5%)	0.69
16:00	2 (4.3%)	1 (3.9%)	0.71	3 (6.4%)	2 (7.7%)	0.59
20:00	3 (6.4%)	1 (3.9%)	0.55	10 (21.3%)	4 (15.4%)	0.39

Data are shown number (%).

**Table 3:** Influence of clinical factors on circadian variability of platelet reactivity according to a one-dimensional mixed model with random effects.

Clinical Variable	VASP				Multiplate			
	Value	SE	*p* Value	R^2^	Value	SE	*p* Value	R^2^
Ticagrelor vs. prasugrel	28.23	5.06	<0.00001	0.31	0.07	0.05	0.12	0.04
Age, years	0.20	0.30	0.50	<0.01	0.08	0.18	0.66	<0.01
Female	9.31	6.42	0.15	0.03	−4.37	3.96	0.27	0.02
Body mass index, kg/m^2^	−0.37	0.67	0.58	<0.01	−0.36	0.41	0.38	0.01
Obesity	1.05	6.74	0.88	<0.01	−6.57	4.05	0.11	0.04
STEMI vs. NSTEMI	−5.94	7.16	0.41	<0.01	2.97	4.39	0.50	<0.01
Hypertension	3.93	6.00	0.51	<0.01	−0.94	3.68	0.80	<0.01
Diabetes mellitus	10.62	7.74	0.17	0.03	1.23	4.80	0.80	<0.01
Hyperlipidemia	15.02	8.66	0.09	0.04	1.68	5.41	0.76	<0.01
Current smoker	−5.95	6.15	0.34	0.01	−0.38	3.79	0.92	<0.01
Prior CAD	−1.90	7.59	0.80	<0.01	−0.66	4.65	0.89	<0.01
Prior PCI	2.57	8.83	0.77	<0.01	6.17	5.36	0.25	0.02
LVEF at discharge, %	0.14	0.42	0.74	<0.01	0.16	0.26	0.55	<0.01
Creatinine, mg/dL	−0.05	13.25	0.99	<0.01	2.15	8.11	0.79	<0.01
GFR, mL/min	−0.02	0.15	0.87	<0.01	0.06	0.09	0.48	<0.01
BNP, pg/mL	−0.01	0.01	0.66	<0.01	<0.01	0.01	0.77	<0.01
Uric acid, mg/dL	0.54	2.11	0.80	<0.01	−0.93	1.28	0.47	<0.01
Hemoglobin, g/dL	−1.16	2.41	0.63	<0.01	−3.19	1.43	0.028	0.06
RBC, 10^12^/L	−2.06	6.39	0.75	<0.01	−5.09	3.87	0.19	0.02
HCT, %	−0.67	0.88	0.45	<0.01	−1.06	0.53	0.047	0.06
WBC, 10^9^/L	0.32	0.87	0.71	<0.01	−0.01	0.53	0.99	<0.01
PLT, 10^9^/L	−0.06	0.05	0.27	0.02	<0.01	0.03	0.96	<0.01
MPV, fL	0.28	0.40	0.48	<0.01	−0.52	0.24	0.03	0.07

BNP: brain natriuretic peptide; CAD: coronary artery disease; GFR: glomerular filtration rate; HCT: hematocrit; LVEF: left ventricular ejection fraction; MPV: mean platelet volume; NSTEMI: non-ST-elevation myocardial infarction; PCI: percutaneous coronary intervention; PLT: platelets; RBC: red blood cells; STEMI: ST-elevation myocardial infarction; WBC: white blood cells.

## Data Availability

The data sets used and analyzed during the current study are available from the corresponding author on reasonable request.
